# Presentation and outcome of COVID-19 in HIV patients with high viral loads and opportunistic infections: a case series

**DOI:** 10.4314/gmj.v54i4s.20

**Published:** 2020-12

**Authors:** Yasmine O Hardy, Divine A Amenuke, Kojo A Hutton-Mensah, David R Chadwick, Rita Larsen-Reindorf

**Affiliations:** 1 Department of Medicine, Komfo Anokye Teaching Hospital/School of Medical Sciences, Kwame Nkrumah University of Science & Technology, Kumasi, Ghana; 2 Department of Medicine, Komfo Anokye Teaching Hospital/School of Medical Sciences, Kwame Nkrumah University of Science & Technology, Kumasi, Ghana; 3 Department of Medicine, Komfo Anokye Teaching Hospital, Kumasi, Ghana; 4 Department of Infectious Diseases, South Tees Hospitals NHS Foundation Trust, Centre for Clinical Infections, Middlesbrough, United Kingdom; 5 Ashanti Regional Health Directorate, Ghana Health Service, Kumasi, Ghana

**Keywords:** COVID-19, advanced HIV, clinical outcome, SARS-CoV-2, Ghana

## Abstract

Coronavirus disease 2019 (COVID-19) is especially severe in patients with underlying chronic conditions, with increased risk of mortality. There is concern that people living with HIV (PLWH), especially those with severe immunosuppression, and COVID-19 may have severe disease and a negative clinical outcome. Most studies on COVID-19 in PLWH are from Asia, Europe and America where population dynamics, antiretroviral treatment coverage and coexisting opportunistic infections may differ from that in sub-Saharan Africa. We report on the clinical profile and outcome of three cases of PLWH co-infected with SARS-CoV-2. They all presented with fever, cough and breathlessness and also had advanced HIV infection as evidenced by opportunistic infections, high HIV viral loads and low CD4 counts. The patients responded favourably to the standard of care and were discharged home. Our findings suggest that PLWH with advanced immunosuppression may not necessarily have an unfavourable disease course and outcome. However, case-controlled studies with a larger population size are needed to better understand the impact of COVID-19 in this patient population.

**Funding:**

Not declared

## Introduction

SARS-CoV-2 infection may present with severe manifestations such as acute respiratory distress syndrome, septic shock and respiratory failure; especially in the elderly and those with co-morbidities.^1^ The risk of succumbing to infections, including viral respiratory infections, in people living with HIV (PLWH) is driven by abnormal immune mediated responses as a result of HIV-related immunosuppression.^2^,^3^ This risk is higher in patients with advanced disease.^2^,^3^ Nevertheless, some studies have reported favourable outcomes of COVID-19 in PLWH.^4^,^5^ To the best of our knowledge most of these studies are from well-resourced settings^4^–^6^ and the findings may vary from reports from sub-Saharan Africa due to differences in population dynamics, co-existing opportunistic infection burden and antiretroviral therapy (ART) coverage. In a bid to help fill this research gap, we describe the presentation and outcome of three cases of PLWH with advanced HIV disease at the Highly Infectious Isolation Unit (HIIU) of the Komfo Anokye Teaching Hospital in Kumasi, Ghana. They were referred from peripheral facilities and had confirmed COVID-19 infection by SARS-CoV-2 polymerase chain reaction on nasopharyngeal swabs.

## Case Reports

### Ethical approval and patient consent

Ethical approval (reference no. KATH-IRB/AP/067/20) was obtained from the Institutional Review Board of the Komfo Anokye Teaching Hospital.

### Patient 1

A 53-year-old ART naive, HIV positive man (time of diagnosis unknown) with Kaposi's sarcoma was admitted at the HIIU.

His complaints were fever and cough of two weeks duration and breathlessness of a day's duration. On systemic review he admitted to sore throat, breathlessness on exertion, chest pain, dysphagia and odynophagia. On examination, he had oral candidiasis, was afebrile (37.3°C) and respiratory rate was 28 cycles/min. Oxygen saturation (SpO_2_) was 92% in room air and rose to 98% on intranasal oxygen at 5L/min. Glasgow Coma Score was 14/15.

His chest X-ray revealed patchy, ground-glass opacities in the left lung field mostly in the middle zone with superimposed consolidation on the opacities. His electrocardiogram (ECG) was normal with a corrected QT interval (QTc) of 0.31 sec. Full blood count revealed moderate hypochromic, microcytic anaemia. Hypoalbuminaemia was noted in the liver panel. CD4 count was 78 cells/microliter and HIV viral load 27,210 copies/ml (Table 1).

**Table 1:** Laboratory finds of the patients

	PATIENT 1		PATIENT 2		PATIENT 3		
Laboratory parameters	Baseline result	Day 12	Baseline result	Day 5	Baseline result	Day 5	Day 10
**Haemoglobin (11.5–16.0g/dl)**	9.3	8.7	7.1	11	7.7	8.5	
**Mean corpuscular volume (78.0–100.0 fl)**	67.6	64.4	76.7	71.8	72.2	73.1	
**Mean corpuscular haemoglobin (26.0–34.0pg)**	22.6	22	27.1	25.6	26.5	25.4	
**White blood cell (4–10x10^3^/microlit)**	4.46	6.67	2.62	8.64	3.96	3.1	
**Neutrophils (1.5–6.6 x 10^3^/microlit)**	1.8	4.4	2.96	7.4	3.15	1.6	
**Lymphocytes (0.8 – 4 x10^3^/microlit)**	2.09	1.68	1.8	0.87	0.51	1.19	
**Platelet count (140–440 x10^3^/microlit)**	374	187	44	87	100	115	
**Alanine transaminase (0–40 U/L)**	19.4	26.3	18.9	27.1	60.5	50.9	62.6
**Aspartate transaminase (0–41 U/L)**	51.1	27.3	22.1	23.5	73.1	276.9	69.9
**Albumin (35–52 g/L)**	22.9	25.1	20.5	22.7	30.4	27.7	28.9
**Urea (2.5–8.3mmol/l)**	5.1	2.4	1	2.6	27.4	25.6	6
**Creatinine (44–106 micromol/l)**	33	65	69	61	545	382	88
**HIV antibodies**	Positive (type 1)		Positive (type 1)		Positive (type 1)		
**CD4 count (cells/microlit)**	78		156		16		
**Viral load (copies/ml)**	27,210		200,008		9,838,407		

He was treated with oral hydroxychloroquine 200mg t.i.d., fluconazole 150mg daily, prophylactic dose (960mg daily) of co-trimoxazole, tenofovir/lamivudine (one tablet daily) and lopinavir/ritonavir two tablets b.d.; 40mg daily of zinc, multivitamins one tablet b.d. and 1g daily of Vitamin C. He was put on a nutritious diet and psycho-social counselling was provided by a psychiatrist.

On day 4, his SpO_2_ was 98% on room air and he was weaned off oxygen. On day 7 most of his presenting symptoms had subsided and his GCS was 15/15. He continued to clinically improve. An ECG done following completion of hydroxychloroquine on day 10 showed elevation of QTc to 0.5 sec; patient was, however, asymptomatic. Two repeat PCR tests for SARS -CoV-2 RNA were done (on days 11 and 12) on nasal wash samples and rectal swab taken 24 hours apart. His PCR results came out negative on day 17 and he was discharged on that day to continue ART care on outpatient basis. He has since been reintegrated into his community by the District Health Management Team and is doing well.

### Patient 2

A 64-year-old woman who had defaulted her ART for about 9 months presented with a 3-month history of recurrent cough productive of yellowish sputum, fever, breathlessness, night sweats, weight loss, and dizziness. She was pale, cachectic, dehydrated and hypotensive (blood pressure of 69/49mmHg). SpO_2_ was 98% on room air.

Chest X-ray showed tracheal deviation to the right, decreased lung volume and lower zone consolidation on the right with compensatory hypertrophy on the left. These findings were suggestive of pulmonary tuberculosis.

A right apical cavity with a mass within suggestive of a fungal ball was also evident. Sputum GeneXpert was negative for mycobacterium tuberculosis. Her ECG was normal with a QTc of 0.42 sec. Blood workup yielded anaemia, thrombocytopaenia and hypoalbuminaemia (Table 1). Escherichia coli, sensitive to gentamycin, was isolated on urine culture.

On day 2, she developed symptoms of anaemia and was transfused with 3 units of blood with improvement of symptoms. Blood pressure rose to 109/74mmHg. She was given intravenous fluids, intravenous gentamycin - 80mg t.i.d., oral hydroxychloroquine 200mg t.i.d., itrazonazole 200mg b.d., three tablets daily of a fixed dose combination of isoniazid, rifampicin, pyrazinamide, ethambutol (HRZE), 100mg daily of pyridoxine, prophylactic dose of co-trimoxazole, multivitamins (1 b.d.), 40mg daily of zinc, 1g daily of Vitamin C and a well-balanced diet. Psychosocial support was offered, and an ART counsellor provided adherence counselling.

On day 7 she developed bilateral cellulitis of her lower limbs and intravenous amoxicillin/clavulanic acid (1.2g t.i.d.) and analgesics were started. On day 10 her symptoms had subsided with marked improvement in the cellulitis so intravenous amoxicillin/clavulanic acid was changed to oral 1000mg b.d.

PCR tests for SARS -CoV-2 RNA (nasal wash and rectal swab) done on days 11 and 12, after completion of hydroxychloroquine, were negative. Repeat ECG showed a QTc of 0.49 sec. On day 14 of taking HRZE she was initiated on a fixed dose combination of tenofovir, lamivudine and efavirenz (1 nocte). The patient was discharged on day 17 to continue outpatient TB/HIV care.

### Patient 3

A 28-year-old newly diagnosed HIV positive male was admitted at HIIU with a 2-week history of cough productive of whitish sputum, fever, weight loss, drenching night sweats, anorexia, fatigue and palpitations. On examination he was febrile with a temperature of 39.2°C, pale and moderately dehydrated. There were matted axillary lymph nodes approximately 3.5cm × 3.5 cm which were non-tender and mobile. He had tachycardia at a rate of 116beats/min and hypotension with a blood pressure of 79/57mmHg. His SpO_2_ on room air was 98%. A chest X-ray revealed bilateral, peripheral, ground- glass opacities in the middle and lower zones on the left suggestive of COVID-19 infection. There was sinus tachycardia on ECG.

Laboratory investigations (Table 1) showed microcytic anaemia, elevated alanine and aspartate transaminases, hypoalbuminaemia, elevated urea and creatinine levels with increased urea/creatinine ratio suggestive of an acute kidney injury likely from dehydration. GeneXpert of the sputum done on day 2 detected mycobacterium tuberculosis. He was given intravenous and oral fluids, oral hydroxychloroquine 200mg t.i.d., prophylactic dose of co-trimoxazole, antituberculous medications [4 tabs of a fixed dose combination of rifampicin, isoniazid, pyrazinamide and ethambutol (HRZE)], pyridoxine 100mg daily, multivitamins 1 b.d., 40mg daily of zinc, paracetamol 1g t.i.d and 1g daily of Vitamin C. A nutritious diet plan was instituted. A qualified psychiatrist provided psycho-social support.

His temperature subsided on day 3. On day 5, his liver enzymes increased with the AST rising to over 5 times the upper limit of normal (Table 1). He however had no symptoms or signs of liver disease. HRZE was suspended and he was given a hepato-protective drug. His liver panel on day 10 showed significant improvement in liver enzymes. HRZE was re-started at a lower dose of 2 tablets daily and later (day 14) scaled up to 3 tablets after checking the liver enzymes.

The patient's clinical picture improved gradually. Nasal wash and rectal swab samples were taken for SARS - CoV-2 RNA tests following completion of hydroxychloroquine and the result came back negative. ECG was also normal. He was discharged on day 18 to continue TB therapy and start ART on outpatient basis.

## Discussion

There is still some debate about the clinical outcomes of PLWH with COVID-19 co-infection. Some authors reported that PLWH did not have more severe disease or worse outcomes compared to their HIV negative counterparts.^4^–^7^ However, their cohorts were mostly patients with controlled HIV on ART and this may have influenced their findings.

Yang et al in a case report of an HIV patient with opportunistic infection, CD4 count of 21 cells/mm^3^ and COVID-19 documented that the patient was asymptomatic, had normal chest CT scan findings and a positive outcome.^8^ The HIV patients in our case series similarly had low CD4 counts, opportunistic infections - Kaposi's sarcoma, pulmonary tuberculosis and fungal infectionand were also virally unsuppressed. None of them had a severe clinical course or fatal outcome.

The favourable clinical picture observed in our case series and the aforementioned studies is speculated to be as a result of the protection against the cytokine storm conferred by the dysfunctional cellular immune response evident in PLWH.^9^ This response hinders the release of cytokines and other inflammatory markers so preventing pronounced inflammation and consequent lung and multi-organ damage.^10^,^11^

A large, population based, cohort study from South Africa however reported poor outcomes with an increased mortality from COVID-19 in PLWH regardless of viral suppression.^9^ In comparison to the patients in our series, these patients had other comorbidities, notably diabetes mellitus and hypertension, which may have contributed to the undesirable outcomes. Karmen-Tuohy et al^5^ reported on a cohort with similar co-morbidities as those in the South African study but in the latter case the patients had tuberculosis in addition which may have contributed to the unfavourable outcomes.

All patients received hydroxychloroquine as part of the standard of care with good response and no record of arrhythmias.

Patient 2 was managed with anti-TB medication in addition with clinical improvement although GeneXpert did not detect mycobacterium tuberculosis. Empirical therapy was given on the basis of a suggestive clinical picture and chest X-ray findings because despite its high sensitivity, GeneXpert may give a false negative result in 14–30% of cases as reported in other studies.^12^,^13^

## Conclusion

In conclusion, our reports showed a favourable disease course and outcome of COVID-19 in three PLWH with advanced HIV in Ghana. This implies that this population may not have a more deleterious COVID-19 course or outcome compared to their HIV negative counterparts. However, this is a case series involving only 3 patients and the findings should be interpreted with caution. More in-depth studies are needed to further elucidate the impact of COVID-19 on morbidity and mortality in PLWH with advanced immunosuppression.
